# Alcohol use during pregnancy and motherhood: Attitudes and experiences of pregnant women, mothers, and healthcare professionals

**DOI:** 10.1371/journal.pone.0275609

**Published:** 2022-12-01

**Authors:** Katalin Ujhelyi Gomez, Laura Goodwin, Anna Chisholm, Abigail K. Rose

**Affiliations:** 1 Department of Primary Care and Mental Health, University of Liverpool, Liverpool, United Kingdom; 2 Division of Health Research, Lancaster University, Lancaster, United Kingdom; 3 Department of Psychological Sciences, University of Liverpool, Liverpool, United Kingdom; 4 School of Psychology, Liverpool John Moor University, Liverpool, United Kingdom; Monash University, AUSTRALIA

## Abstract

Alcohol is the most used substance by women of childbearing age. Alcohol exposed pregnancies can have serious consequences to the fetus, and the UK has one of the highest rates of drinking during pregnancy. Alcohol use during motherhood is also a public health concern, linked with potential harms to the woman and child. This qualitative study investigated the attitudes and experiences of pregnant/parenting women and healthcare professionals regarding maternal drinking. A semi-structured focus group and interviews were conducted in the North West of England with pregnant women, mothers, and healthcare professionals. Quantitative measures captured demographics, alcohol use, and screened for mental ill-health for pregnant women and mothers. Reflexive thematic analysis was used to analyse narratives. Findings revealed that most participants believed avoiding alcohol during pregnancy is the safest option. However, some pregnant women and mothers stated that there was insufficient evidence to demonstrate the harms of low-level drinking and that abstinence guidelines were patronising. All participants reported that low-level drinking during motherhood was acceptable. Heavy drinking was believed to pose serious harm during pregnancy and motherhood to the baby and mother, in addition to damaging relationships. Strong motives were revealed for choosing and avoiding to drink, such as coping with the difficulties of motherhood and parental responsibilities, respectively. Contradictions were found across quantitative and qualitative self-reports of consumption, reflecting potential underreporting of alcohol use. Additionally, drinking levels were discussed in extremes only (low/heavy) without considering ‘grey area’ drinking. Clear, consistent advice and guidelines are needed to support women in reducing their alcohol use during pregnancy and motherhood. These should include the unique potential risks regarding maternal drinking, and the harm attributable to non-clinically dependent alcohol use. The maternal participants in this study were middle-class, therefore, research is needed to capture the views and experiences of women of all socioeconomic backgrounds.

## Introduction

Within the UK, the decreasing gender gap in alcohol use is being driven by an increase in women’s drinking and, worldwide [1], nearly one third of diagnosed alcohol use disorders are among females [2]. This has implications for increased alcohol harm as women are more susceptible to the negative impact of alcohol both psychologically [3] and physically [4]. For example, women with alcohol use disorders have a higher likelihood of suffering from comorbid common mental health disorders (including anxiety and depression), than men [5]. Women also metabolise alcohol differently from men, reaching higher and longer lasting blood alcohol concentrations [5]. This can contribute to the development of more severe problems at younger ages and lower drinking levels than men, including cirrhosis of the liver, heart disease, and neurotoxicity [6]. Alcohol is also a risk factor for typically female diseases, such as breast cancer [3].

Importantly, women’s alcohol use is also associated with key secondary harms and alcohol is the most commonly used substance by women of childbearing age [2]. The UK has one of the highest international rates of drinking during pregnancy (41.3–75%), which translates to one of the highest prevalence rates of fetal alcohol spectrum disorder (FASD) (3.2%; [7]) and other child-related developmental and behavioural problems [8]. It has been suggested that continued alcohol consumption during pregnancy may be the result of a lack of awareness around the severity of risks and the specific details of the effects, in addition to conflicting advice from different sources, regarding whether any ‘safe’ drinking limits exist during pregnancy [9]. For example, guidance by the UK Chief Medical Officer was revised in 2016 advising pregnant women to abstain due to the lack of evidence for a safe drinking level. These changes were, however, not introduced to the National Institute of Health and Care Excellence (NICE) guidelines immediately and for the following three years, two guidelines with differing advice were in place. However, although knowledge and attitude are key influencers of behaviour [10], awareness alone is not sufficient to change behaviour [9]. This is suggested by the finding that women with higher levels of education, so perhaps higher levels of awareness, are more likely to consume alcohol during pregnancy [11].

Transition into motherhood can be associated with reduced alcohol use, attributable to changing social norms and increasing responsibility [10]. Yet it can also be a risk factor for increased drinking due to enhanced levels of stress and social isolation [12]. Research has demonstrated that although many women report reduced alcohol use during pregnancy and in the first 12 months postpartum, this protective effect declines after one year and alcohol use returns to pre-pregnancy levels by 5 years (i.e., UK school age) [13]. There is growing narrative that mothers (especially of young children) are using alcohol as a reward or to cope with isolation and the stresses of parenthood [12]. Other possible reasons why mothers may drink include pressures of perceived social norms; being unaware of, or discounting, health risks; and coping with mental health problems and trauma [12, 14]. For example, psychological birth trauma, a subjective experience, has recently been associated with increased drinking in mothers [15]. Overall, drinking during motherhood may place women and their children at heightened risk for long-lasting adverse physical (e.g. [16, 17]), mental (e.g. [18, 19]), and behavioural (e.g. [20]) consequences. In the UK, an estimated 5%-18% of mothers with children between the ages of 9–12 months and 14 years drink at increased risk [21]. Additionally, around 1 in 17 UK children, up to 5 years of age, live with a mother with alcohol misuse recorded on primary care health records [21].

Importantly, drinking does not have to reach clinically-dependent levels to be harmful [22, 23], yet many estimates come from treatment cohorts. This suggests that the real negative impact of maternal alcohol use is very likely underestimated. Often mothers will not report their alcohol use accurately to healthcare professionals due to stigma, fear of disclosure consequences [24] and wanting to come across as a ’good’ parent [25].

Given the significant potential primary and secondary harms associated with maternal drinking (i.e. drinking during both pregnancy and motherhood), we need a better understanding of the attitudes and experiences of pregnant women and mothers, and how this compares to the views of relevant healthcare professionals (e.g. midwives, general practitioners (GPs)). This understanding should help overcome the current limitations we have in providing appropriate and effective support in helping pregnant women and mothers to reduce their alcohol consumption [26]. This study looked at the attitudes and experiences of pregnant/parenting women and healthcare professionals regarding alcohol consumption during pregnancy and motherhood. The study addressed the following research questions: 1) Why do women drink alcohol and why do they abstain during pregnancy and motherhood and 2) What are the perceived harms of alcohol consumption during these periods? 3) How are the attitudes of women and healthcare professionals different or similar in terms of alcohol use?

## Methods

### Participants and research team

The study was conducted in the North West of England and involved pregnant women, mothers with dependent children (defined as children under 18 years of age living at home), and relevant healthcare professionals. To be eligible to participate, participants had to be 18 years or older, be pregnant and/or the mother of one or more children of dependent age and/or a healthcare professional, be a fluent English speaker, and be able to provide informed consent. If a woman was pregnant and had children of dependent age, she was grouped with the pregnant participants. Healthcare professionals were recruited to represent the key health contacts for women during pregnancy and motherhood (midwives, GPs) and for women experiencing alcohol use problems (substance misuse practitioners). All individuals who requested to participate were included.

The study received ethical approval from the University of Liverpool Central University Research Ethics Committee. Participants of this study provided written informed consent.

The research team included female researchers with PhD qualifications. The researchers collecting and analysing data had no prior relationship with the participants of the study. The principal investigator (AR, senior lecturer) has substantial experience of researching alcohol use and wellbeing, and the research associate (KUG) has expertise in qualitative methodologies and analysis. The co-authors (LG and AC, senior lecturers) are experienced academics in the field of mental health and qualitative research methods, respectively. The focus group and majority of interviews were conducted by KUG, with three interviews with mothers conducted by two psychology master’s students (after receiving training and supervision from AR and KUG).

### Measures

Multiple qualitative methods investigated the attitudes and experiences of women and healthcare professionals (N = 21) to accommodate availability. This included a semi-structured focus group with midwives and a substance use worker (N = 4) and one-to-one semi-structured interviews with general practitioners (N = 3), pregnant women (N = 6), and mothers (N = 8). The schedule for the interviews and the focus group were consistent, but tailored to the participant group (i.e., pregnant women, non-pregnant mothers, healthcare professionals. See S1 and S2 Files. The schedules comprised open questions designed by the research team and reviewed by an independent academic peer with qualitative expertise. They aimed to elicit participants’ attitudes around drinking during pregnancy and during motherhood separately, including questions around the risks, benefits, and long and short-term effects of drinking, and the motives for and against alcohol use during these periods of their lives. The questions for healthcare professionals were worded slightly differently inquiring about the alcohol consumption of mothers and pregnant women in general rather than about themselves.

Pregnant women and mothers also completed basic well-established quantitative measures to provide a more detailed picture of participant variables which may influence drinking behaviour. Demographic information was collected including age, ethnicity, relationship status, education level, occupation, working status (i.e., maternity leave), annual household income, and residing area. Participants also provided information regarding the number and age of their children, and whether they were currently pregnant and if so their due date. All quantitative scales were self-report measures.

### Alcohol use and mental health

#### Alcohol Use Disorders Identification Test (AUDIT)

The 10-item AUDIT [27] was used to assess drinking behaviour providing an assessment of low-risk (AUDIT 1–7), hazardous (AUDIT 8–15), and harmful alcohol use (AUDIT 16–19), and dependence (AUDIT 20+). A score of 7 or more in women indicates the likelihood of hazardous or harmful drinking [28]. Mothers (who were not currently pregnant) were asked if they drank during pregnancy and if so the first and second items of the AUDIT were used to ask about frequency and quantity of alcohol use, in addition to a question about the type of drink usually consumed.

#### PHQ-2 and GAD 2

Depression was measured by the short 2-item version of PHQ-9 using the first two items of the original scale inquiring about frequency of depressed mood and anhedonia over the past two weeks. Scores range between 0–6 with a score of three or higher indicating likely depressive disorder [29]. GAD 2 measured anxiety by using the first two items of the GAD 7. Scores range between 0–6 where a score of three or above indicates possible generalised anxiety disorder [30].

#### Birth experience and trauma

Mothers and pregnant women who had previously given birth reported their subjective individual experience regarding birth trauma by responding to the question “*Irrespective of whether you sought help or have people around you that understand your experience*, *do you think you have experienced a traumatic birth*?*”*. Mothers and pregnant women also reported whether they had experienced any other traumatic life event (not related to childbirth) using the initial screening question of the Primary Care PTSD Screen for DSM-5 [31].

### Procedure

The study was advertised on social media and through distributing posters/flyers at public establishments in advantaged and disadvantaged neighbourhoods. Pregnant women and mothers were recruited from a local university, children centres, and family friendly pubs. Healthcare professionals were recruited from the local women’s hospital (midwives), healthcare centres (GPs, working with patients from a range of socioeconomic backgrounds), and a drug and alcohol service (substance misuse worker) by email invitation.

Prior to the interviews and the focus group, participants were provided with a Participant Information Sheet and gave their written informed consent. Pregnant women and mothers completed the quantitative measures (alcohol use, mental health, birth experience) and demographic information was collected from all participants. All data were anonymised to protect participant identity by assigning a letter and a number to each participant: M# = mother, P# = pregnant woman, MW# = midwife, SMP# = substance misuse practitioner, GP# = general practitioner.

The focus group and most interviews took place on campus, with some conducted in the participants’ home and healthcare centres. One interview was conducted at the local library as per the participant’s request. To accommodate the availability of healthcare professionals, a focus group was conducted first with the midwives and a substance misuse practitioner, which lasted approximately 70 minutes. During the focus group, a researcher, not involved in the project, was present to provide support if needed and took notes. This was followed by three separate interviews with GPs, who were unable to attend the focus group due to work commitments, providing complimentary material. Making the pragmatic decision of using focus groups in combination with interviews has been previously recognised [32]. Interviews were 20 to 60 minutes long. One interview with a GP was repeated due to the failure of technical equipment during the first attempt. Where necessary, field notes were taken by the interviewer after interviews.

Data saturation in the form of ‘informational redundancy’ [33, 34], where further data does not yield new information [35], is not a goal in reflexive thematic analysis [36, 37] as data saturation is not particularly useful or theoretically coherent. Rather, the notion of information power, whereby the relevance of the information collected rather than the number of participants, may provide a better way of considering data samples [36]. Therefore, data collection did not focus on saturation.

### Data analysis

The focus group and interviews were audio recorded and transcribed verbatim. The Nvivo 12 qualitative software programme was used as an aid in data analysis. The researcher conducting the data analysis (KUG) has training and experience of content and thematic analysis of data. Transcriptions were coded and categorised using content analysis and reflexive thematic analysis [37, 38] where data was sufficient. The categories were arranged into major inductive themes based on the study aim to explore participants’ alcohol-related attitudes and experiences, specifically, 1) to determine motives to drink and to reduce drinking or abstain during pregnancy and motherhood and 2) to identify perceived harms of maternal alcohol use. Themes were reviewed and refined further and discussed with AR and AC. Data obtained was analysed independently by participant groups.

Different data collection methods were used for healthcare professionals. Midwives and a substance misuse practitioner attending a focus group, and GPs attended one-to-one interviews at a later time. All healthcare professionals were requested to answer the same questions and responses were consistent, therefore the data was deemed adequate for combined analysis.

## Results

### Participant characteristics

A total of six pregnant women, eight mothers, and seven healthcare professionals participated. The majority of pregnant and parenting women were white British (N = 10, 71.43%), 30 years and older (N = 12, 85.71%), completed a bachelor’s degree or above (N = 12, 85.71%) with a household income of higher than £31000 (N = 10, 71.43%). All participants, but one GP, were female. Most pregnant women and mothers were co-habiting or married (N = 13, 92.86%). Half of the mothers (N = 4, 50%) had experienced childbirth as traumatic and half of the pregnant (N = 3, 50%) women had non-birth related traumatic life experience(s). Only one pregnant woman, but most mothers (N = 7, 87.5%) reported any alcohol consumption. All pregnant participants scored zero on the AUDIT scale, however, the reporting of one participant (P2) was difficult to interpret due to unclear and contradictory responses. The score of most mothers demonstrated lower risk drinking (≥7) in general with two participants drinking during breastfeeding (6–9 units/week). Anxiety and depression levels were low across participants, except for one pregnant participant screening positive for probable depression. Healthcare professionals included three midwives, one substance use worker, and three GPs with substantial professional experience. The majority of professional participants were from a white ethnic background and one of them was a male. Four of the healthcare professionals had children themselves. Although they were interviewed in their capacity as a healthcare professional, their experiences as mothers permeated their responses. Full participant characteristics are included in S1 Table.

Upon analysing the narratives, substantial similarity was discovered between the themes generated from the different participant groups with differences appearing in terms of views of healthcare professionals on alcohol consumption during pregnancy (S1 Fig). To avoid repetition and to clearly demonstrate the similarities and differences between the views of women and healthcare professionals (aim 3), in this section themes from all groups are presented together with any differences highlighted in terms of themes or theme content. Results are reported separately for maternal periods. S1 and S2 Figs provide a graphical map of the generated themes.

### Alcohol consumption during pregnancy

The attitudes and experiences of women and healthcare professionals are presented in three overarching themes in relation to alcohol consumption during pregnancy: 1) *To drink or not to drink*; 2) *Drinking during pregnancy is associated with high risk*; and 3) *Strong behavioural motives*.

#### Theme 1: To drink or not to drink?

Women’s attitudes were split based on whether or not they believed drinking was acceptable during pregnancy. Most mothers and pregnant women reported that drinking alcohol during pregnancy should be strictly limited or should not occur as it was the safer option. With the exception of one, these women did not drink once they were aware of their pregnancy or reported to have drunk the occasional ‘sip’ or ‘once or twice’ during pregnancy. The remaining mothers and pregnant women believed low level drinking was acceptable based on the lack of convincing evidence to show the harmful effects of low-level drinking. These women felt judged, stigmatised, and their autonomy threatened. One pregnant woman believed that low level consumption may not be harmful, although she did not drink herself due to negative experiences of alcohol-related problems in her family. All healthcare professionals reported that they advise women to abstain from alcohol through pregnancy as that is the safest option, however, recognising from their own experiences as mothers that women may find this difficult.

#### Theme 2: Drinking during pregnancy is associated with high risk

Three sub-themes were identified in terms of perceived risks of drinking in pregnancy. Drinking during pregnancy was primarily associated with harms to the fetus in addition to some negative impact on the mother’s psychological and physical health, as well as the potential to have a negative effect on relationships. Healthcare professionals’ narratives closely resembled the data obtained from women.

*Sub-theme 1*: *‘Consequences to Baby’*. Perceived harms of excessive drinking to the baby included developmental issues, birth defects, foetal alcohol spectrum disorder, miscarriage or stillbirth, addiction for the child, neurobiological delays, low birth weight, heart and other organ problems, interference with gestational time, cognitive defects, and behavioural issues later in life.

*Sub-theme 2*: *‘Consequences to Mother’*. Women associated higher level drinking with problems with decision making ability, risky behaviours, hangover, decreased awareness of own well-being, health, and the ability to look after themselves, and difficulties raising an unwell child later on. Physical issues of excessive alcohol intake were reported as heart palpitations, hepatitis, and liver damage, while a range of psychological problems were also mentioned: developing alcohol dependence, lower well-being, increased mental health problems (e.g. mood swings, depression, anxiety), and feelings of guilt and self-blame for problems with the baby. Drinking at low levels and perhaps without harming the baby may also cause feeling uncomfortable and regretful about drinking. Healthcare professionals highlighted the general risks of alcohol consumption without being pregnant that also apply during this period and the additional strain that may be placed on the body by alcohol consumption while pregnant.

*Sub-theme 3*: *‘Damage to Relationships’*. Narratives related alcohol use to potential disrupted family dynamics and isolation. Women felt that family members may have differing views on drinking during pregnancy and even a judgmental attitude towards the mother, leading to disagreements and friction. Having an unhealthy child could put strains on the entire family due to, for example, the child’s behavioural problems and may lead to family members feeling angry and blaming the mother for any negative outcomes.

#### Theme 3: Strong behavioural motives

Two sub-themes were generated to demonstrate drinking- and abstinence or reduction-related motives.

*Sub-theme 1*: *Motives to drink*. Five motives to drink were identified. Pregnant women and mothers reported that women drink alcohol during pregnancy to cope and relax, because they may not be aware of the serious consequences, and to gain a sense of normality. In addition to coping and awareness about consequences, healthcare professionals identified some social aspects of drinking and the challenges of changing health behaviour. The most mentioned reasons for drinking included coping and social aspects.

‘Drinking as Coping Strategy’: Some women drink alcohol to relax, aid sleep, ease stress, deal with their emotions, and ‘medicate’ psychological issues. Moreover, an existing alcohol dependence and/or mental health problem may be the reason why women are unable to stop when they get pregnant. Healthcare professionals suggested that women may also turn to alcohol to rebel against an unwanted pregnancy or to cope with domestic violence.

‘Awareness and beliefs about consequences’: Women may drink being unaware of their pregnancy. Others may not appreciate how severe potential consequences may be due to misconceptions, lack of clear evidence and inconsistent advice, which may provide a rationalisation for women who choose to drink.

‘Drinking Provides Normality’**:** Data from women suggest a social aspect to drinking. The focus, however, is primarily on feeling normal; continuing with usual activities as much as possible without losing lifestyle choices.

‘Social Aspects’: Although healthcare professionals’ narratives included discussion around normality, they focused more on alcohol having a major, engrained role within the UK’s socialising culture. Pregnant women may feel left out if they can’t enjoy a drink with others at social gatherings, such as weddings. They desire ‘being normal’ and to fit in with the crowd as pregnancy tends to lead to major changes in one’s life in terms of appearance and expected behaviour. Drinking at social events may also help to feel more relaxed.

‘Challenges of changing health behaviour’: This was an additional theme that emerged for health care professionals. When a woman becomes pregnant, she is expected to change her behaviour and entire lifestyle all at once to protect the baby and have a healthy pregnancy. This may feel overwhelming, particularly at the very early stages of pregnancy when women are getting used to the idea of being pregnant. They may struggle to give up behaviours they are accustomed to, such as having a drink at the weekends. As the pregnancy progresses and attachment to the fetus develops, it becomes easier to adhere to these expectations of lifestyle change.

*Sub-theme 2*: *Motives against drinking*. Reasons reported by women for avoiding drinking is presented within two sub-themes describing the negative consequences to baby and mother, and women’s personal preference to abstain. In addition to a preference to abstain, healthcare professionals focused on the consequences of drinking to the baby and mothers’ fear of being stigmatised for which a separate theme emerged.

‘Negative Consequences’: Participants argued strongly against drinking due to its damaging effects on the fetus (fetal alcohol spectrum disorder, developmental defects) and a desire to have a healthy pregnancy. This reflects the narratives of healthcare professionals, some of whom were also mothers with similar opinions. This knowledge was reported to be the result of receiving health advice from professionals to avoid alcohol owing to the lack of evidence on safe-level drinking. Women reported physical safety and health as a reason to avoid drinking alcohol, in addition to the potential feelings of guilt of harming the baby. Finally, social stigma appears to influence drinking behaviour with social norms dictating not to drink during pregnancy.

‘Personal Preference’: In line with previous research [39], women who did not drink before pregnancy, would not drink while pregnant. Some women may also develop a distaste to alcohol or often feel tired and poorly. Others may accept professional advice not to drink and they consciously want to do their absolute best to have a healthy pregnancy.

‘Fear of Stigma’: While women reported several negative consequences that may affect them, stigma and judgemental attitudes emerged as a full theme from the narratives of healthcare professionals as a motive against drinking. Healthcare professionals include the negative consequences highlighted by women, as risks of drinking as opposed to reasons for not to drink. Themes, sub-themes, and associated example quotes are presented in Table 1.

**Table 1:** Alcohol consumption-related views and experiences of pregnant women, mothers, and healthcare professionals during pregnancy.

Main themes	Sub-themes	Example quote
**To drink or not to drink**		
	***Low level drinking is acceptable***	*“I can’t speak about other people*, *my personal reason is if I want to have a glass of wine I’ll have a glass of wine but as I say that’s been very seldom”* (P2)
		*“I think you’d need to convince people that it was dangerous and it would need to feel like it’s not propaganda…that if it was harmful that the harms outweighed the benefits”* (M6)
		*“…all the evidence showed that…abstaining totally wasn’t completely necessary”* (M2)
		*“Obviously I think you have to be responsible…So I wouldn’t condone sort of regular binge drinking or erm even regular sort of erm 1 or 2 drinks a night*, *but I don’t see a problem with having the odd drink here and there throughout pregnancy…I think a lot of the time we’re made to feel bad about it*.*”* (M2)
		*“It tends to be demonized and people…feel that they’re ok to pass judgement on you I guess erm and that’s not acceptable*, *like you become public property for people to make a comment on and it’s like I don’t think so like I’m not public property*.*”* (M6)
	***Better to be safe***	*“Just don’t think it was worth the risk knowing that it’s going straight to the baby*.*”* (M1)
		*“I think the benefit is zero because like the safest amount it’s difficult to measure as well because you know how much would be too much for the baby and it’s kind of like difficult risks so that’s why it recommended zero and again you know in general people drink too much so it’s wise advice not to drink”* (P6)
		*“So even though professionally I will tell women you know there’s no safe limit*, *the advice is to avoid alcohol at all cost in pregnancy*, *I talk about erm the risk to the baby but I also have an awareness personally that it then restricts you quite a lot*.” (MW1)
**Drinking during pregnancy is associated with high risk**		
	***Consequences to baby***	*“…addiction of the child but also their health*, *it’s like smoking obviously smoking is bad and they say smoking’s bad so your child will be born quite small*, *they’ll have health issues…”* (P2)
	***Consequences to mother***	*“I mean if you know that you’ve damaged your child because of something that you didn’t need to do*, *that’s that must just be awful*.*”* (GP2)
	***Damage to relationships***	*“I guess it depends on the amount that you’d be drinking and then how people would view your pregnancy and view you because another thing is that you know you’re viewed as very much like a home for another being you’re not you*, *stop being like just yourself*. *So people are very judgemental about what you chose to do no matter whether you’re drinking or not drinking*, *or what you’re eating*, *what you’re not eating*.*”* (P5)
**Strong behavioural motives**		
***Motives to drink***		
Women	*Drinking as Coping Strategy*	*“I think depression*. *I don’t drink but I think a few people I have come across have been depressed*. *Some first-time mums*, *they might not really know what to do*, *and when they are depressed*, *confused*, *worried they find solution or comfort from alcohol which is not a good idea you know*?*”* (M8)
	*Awareness and Beliefs about Consequences*	*“*…*every now and then there are some headlines and some stories saying…it’s not as bad as we thought…and people generally stop trusting science and recommendations and guidelines…whenever there is anything that opens a little window to it I think people will gladly take it as they would rather have a couple…”* (P3)
	*Drinking Provides Normality*	*“We went for a very nice meal to a … 2-Michelin star restaurant the other day and it was a very special occasion so I had a small glass of wine*.*”* (P2)
Healthcare professionals	*Drinking as Coping Strategy*	*“…coping for all sorts of reasons for mental health*, *for general stress*, *going through a bad time*, *could be something like domestic violence*, *it could be anything really*
	*Awareness and Beliefs about Consequences*	*“‘Oh well yes I had a glass of Guinness because I’ve got low iron’*, *we get that a lot*, *‘my iron is low so I’ve had a Guinness’*, *no it’s not how it works*.*”* (MW2)
	*Social Aspects*	*“…you feel left out*, *you don’t feel part of the normal circle*, *you look different to people and you might not be happy with how you look*. *So you just want normality*, *you want to keep some things the same and if that’s drinking and socialising is one of them that will be the benefit*.*”* (MW3)
	*Challenges of changing health behaviour*	*“Someone’s just handed you a stick and said you’re pregnant change all your life you know you’re drinking habits*, *your smoking*, *your food*, *don’t eat this don’t eat that there you go and you don’t feel connected to the baby yet at that point*.*”* (MW1)
***Motives against drinking***		
Women	*Negative Consequences to baby and mother*	*“It’s kind of the information out there about the impact that you know that it goes you know the placenta doesn’t stop it goes straight to the baby any alcohol that you drink*. *So I think it’s just concerns over that”* (M1)
		*“I think if anything did happen you’d probably feel like there was a dereliction of duty somewhere along the way like you hadn’t done what you were supposed to do again like I mentioned before I already felt a failure”* (M6)
		*“So I think like other people will think of it before you so it’s almost as if that’s what they’re sort of like projecting their views on what’s acceptable and what isn’t on you*…*”* (P4)
	*Personal preference*	*“…if you’ve already got other children who are making you…and feeling sick and poorly I just did not want to have a drink in those first few months”* (M2)
Healthcare professionals	*Consequences to baby*	*“Women are told that it is bad for the baby*, *so they stop because they can and because it’s temporary*, *it’s not like someone saying you have to never drink at all*.*”* (GP2)
	*Fear of stigma*	*“You don’t really want to be a bump sat in a pub with a pint*.*”* (MW1)
	*Personal preference*	*“They just find they can’t drink during pregnancy*, *it makes them feel ill and they don’t actually want to have a drink*.*”* (GP1)

P = pregnant woman; M = mother; GP = general practitioner; MW = midwife

### Alcohol consumption during motherhood

With regard to alcohol consumption during motherhood, three overarching themes emerged representing the attitudes and experiences of women and healthcare professionals: 1) *Low-level drinking is acceptable*; 2) *Excessive drinking poses risks*; and 3) *Strong behavioural motives*.

#### Theme 1: Low-level drinking is acceptable

Women and healthcare professionals believed that drinking alcohol at a low level/in moderation/occasionally is acceptable. However, they felt that drinking to excess and becoming intoxicated in general, and particularly when around their child(ren), could result in negative consequences. With reference to breastfeeding, most women chose not to consume alcohol, with some believing a limited amount was acceptable. However, it is evident that these women may consume up to 9 units of alcohol a week as demonstrated by their scores on the alcohol measures used. Although this is within lower risk guidelines for adults, NCT breastfeeding guidelines recommend no more than 1–2 units up to twice a week [40].

The definition of acceptable drinking level varied: *“A glass of wine with a meal…”* (P2), “*social drinking of a glass of wine here or there”* (P2), *“…a glass of wine every other night and couple of glasses on the weekend…”* (M5). In five of eight of the mothers, contradictions appeared within interview narratives, and when comparing narrative responses to quantitative responses around alcohol use (e.g., AUDIT items).

#### Theme 2: Excessive drinking poses risks

Participants believed that drinking alcohol can: have serious health consequences; result in poor parenting, and; potentially cause damage to relationships.

*Sub-theme 1‘Health Consequences’*. Participants believed that drinking can lead to physical and mental health problems, particularly in early motherhood when women are vulnerable to common mental illnesses (e.g., anxiety, depression). It may also contribute to feelings of guilt if the drinking leads to child neglect. Participants also considered alcohol’s high unhealthy calorie content as important. Physical health issues mentioned include cirrhosis, liver, heart, and kidney damage. There were concerns regarding drinking becoming more regular leading to alcohol dependence, as well as its cumulative effect on one’s personal and work life.

*Sub-theme 2*: *‘Poor Parenting’*. Alcohol consumption may bring about poor parenting, a factor that influences many women to avoid drinking, as will be demonstrated in a later theme *‘Parental Responsibilities’*. However, these risk factors do not necessarily stop all women from drinking. Women mentioned the dangers of alcohol use during the breastfeeding period. Despite potential risks, some chose to drink at a low level as they believe that a small amount of alcohol is not harmful.

There were extensive reports on issues with the child’s physical and emotional needs and safety, such as being physically and emotionally (un)available, having the capacity to look after their children, and the issue of co-sleeping. From a professional perspective, alcohol may raise issues in terms of safeguarding children, such as whether there may be a case of neglect. Drinking was seen as an issue that may lead to emotional and physical unavailability of the mother, negatively affecting the child’s behaviour. Looking after children with a hangover was reported as a factor that may lead to poor parenting. Providing the wrong example was also a concern, which discouraged some women not to drink in front of their children. Although, responsible drinking behaviour was thought to provide a good example of safe drinking in some cases, other children may engage in problematic drinking witnessing their parents’ excessive alcohol use.

*Sub-theme 3*: *‘Damage to Relationships’*. According to narratives, drinking excessively may disrupt the family dynamics and prevent women from performing their role as a mother becoming reliant on the support of others. Participants also related excessive drinking to domestic violence and other conflicts within the family unit due to impulsive and reckless behaviour., and family members disagreeing with drinking around children and/or being concerned about the mother. Dealing with children’s behavioural problems as a consequence of the damage of alcohol on the child’s health may also produce familial issues. Additionally, due to regular and problematic alcohol use, mothers may fail to be emotionally available which will negatively influence the relationship between mother and child, leading to attachment problems.

#### Theme 3: Strong behavioural motives

Two sub-themes were identified that represent motives in relation to alcohol use.

*Sub-theme 1*: *Motives to drink*. Within this sub-theme, two further sub-themes emerged explaining that parenting women use alcohol as a coping strategy and due to its cultural nature as a tool to socialise.

‘Drinking as a Coping Strategy’: Motherhood was described as a stressful and challenging period for women which requires coping strategies. Alcohol is primarily used as an aid to relaxation, stress relief, and sleep. Some women may also struggle with negative emotions and mental health problems due to a lack of support and feelings of isolation and loneliness. Drinking may also occur due to alcohol dependence.

‘Social and Cultural Aspects’: Alcohol was reported to have a significant role in society, where culture supports social activities revolving around drinking and binge drinking. For women, and for mothers in particular, drinking has become the social norm. It provides mothers with the ability to fit in, to have a social life, and to reclaim their identity. Mothers would consume alcohol because they find it enjoyable, a rare opportunity to *“make the most of that night out”* (P4), it is part of their lifestyle, and it provides them with a feeling of normality engaging in activities unrelated to motherhood. Alcohol also helps social participation. In Britain drinking alcohol is viewed as the norm with not participating in drinking being considered *“weird”* (P3). Participants reported that having alcoholic drinks forms a major part of the general culture, but recently, it has also been normalised among mothers and advocated in the media.

*Sub-theme 2*: *Motives against drinking*. Two sub-themes were generated explaining that women would avoid drinking alcohol when their children are young to meet their parental responsibilities and because of personal choice.

‘Parental responsibilities’: The infant’s physical dependence on the mother was mentioned as one of the main factors that stops women from consuming alcohol. Some women are concerned about the transfer of alcohol during breastfeeding and the physical safety of the baby co-sleeping with a parent whose sleep might be affected by alcohol. However, narratives also revealed that in the case of co-sleeping this fear may reduce when mothers have more children and become more experienced in child rearing. Later the focus shifts to the parents’ responsibility to model appropriate drinking behaviour to their children whether it is no drinking or moderate drinking on special occasions and to maintain their children’s perception of them as reliable caregivers. Anxieties about being a new mother are often heightened and there are concerns about being available physically and emotionally for the baby and around the child’s physical safety near intoxicated people. They feel that they want to be in full control when having to attend their children’s needs and to avoid potential feelings of guilt if something goes wrong. Moreover, many women are uncomfortable with the idea of looking after a child with a hangover. One mother felt that a mother drinking was viewed as inappropriate behaviour as opposed to the father having a drink. Pregnant women talked about becoming a mature adult as a result of having children. Interestingly, only pregnant women without children reported that they would lack the opportunity to socialise and consume alcohol due to the changes of circumstances that having a child brings about. Mothering women reported to have found ways of drinking other than during social outings, such as in their home.

One of the pregnant participants (P1) came from a lower socioeconomic background and had been diagnosed with alcohol use disorder (AUD). Her report was inconsistent at times with the narratives of other participants. For example, her reason for abstaining was a fear of losing custody of her children: *“Well it’s not sensible…really isn’t because like my situation because I had a drink and my son got took into care…”*.

‘Personal Preference’: Women chose not to drink as a personal preference in terms of leading a healthier lifestyle, financial considerations, disliking alcohol or being unable to tolerate its effects on the body, and wanting to be mindful of every moment of motherhood. They have less opportunity to socialise which would be their reason to drink. Participants assumed that for those recovering from an alcohol problem, abstinence may serve to avoid relapse. Furthermore, having negative previous alcohol experiences, such as witnessing parental alcoholism, was also reported to be a contributing factor to abstaining. Table 2 includes themes, sub-themes and example quotes.

**Table 2:** Alcohol consumption-related views and experiences of pregnant women, mothers, and healthcare professionals during motherhood.

Main themes	Sub-themes	Example quotes
**Low-level drinking is acceptable**		
		*“It all depends on the situation*, *so if the mother is*, *you know from one end is just having one drink with a friend and her children are being well looked after*, *I don’t see there’s an issue but you have another side of the spectrum where a mother is alcohol-dependent*, *her children are at risk*, *they’re not having their physical*, *mental*, *emotional needs being met*, *and she is herself*, *making herself vulnerable if she is also intoxicated*. *It can be*, *you know*, *drinking in motherhood can be completely fine or completely detrimental to the children*. *It’s just a spectrum*.*”* (GP1)
**Excessive drinking poses risks**		
	***Health consequences***	*“If you’re drinking excessively your mental health is going to duck you know it has an impact on mood”* (M5)
		*“People can easily start drinking especially in the evening or when the kids have gone to bed*, *and they think ‘ oh it’s time for me and I’m gonna relax’ and that’s the way that they kind of quickly relax and that can so easily become a habit and get out of control*, *really I think*. *I’ve seen quite a few women*, *where that’s being the problem and they’re aware of it*.*”* (GP2)
	***Poor parenting***	*“I’m breastfeeding my little boy*, *erm and I know there’s a lot of sort of mixed reviews over whether or not you’re able to drink or not whilst you’re breastfeeding*. *Erm*, *I personally do*, *I have a glass of wine or two each week*, *erm and it’s my understanding I’ve done a lot of reading around it and my understanding is drinking in moderation isn’t harmful when you’re breastfeeding*.*”* (M7)
		*“If the alcohol will become a problem that could affect their you know learning and their support that they get from parents erm maybe they will have less attention*, *maybe they will you know have less help with you know school and generally have worse performance you know that could definitely affect it long term because then you know it will be just dragging behind them for you know their opportunities and the chances they get in life”* (P3)
		*“If the kids there the kids going to see you so it’s going to affect mentally*, *physically and emotionally erm erm yes that its yes just more the child really”* (P1)
	***Damage to relationships***	*“You see families breaking down because of alcoholism*. *You know*, *things that are done when somebody’s drunk*. *You know*, *they can maybe do impulsive*, *reckless behaviour and can break down relationships*, *as well*.*”* (GP1)
		*“Serious implication on bonding with the child”* (M5)
**Strong behavioural motives**		
***Motives to drink***		
	*Drinking as coping strategy*	*“Stress erm a way to unwind cause its incredibly difficult you know when you’re a new mum and like erm I found it really hard a combination of sleep deprivation erm boredom like it’s so boring at times it’s so tedious being a new mum”* (M6)
		*“*…*Like some single parents*, *you know when they are just alone or on their own*, *or the man is not involved in their life they tend to fall into depression*. *So*, *they see the alcohol as a kind of comfort to them…”* (M8)
		*“I think motherhood is a really stressful time for women*, *so they may*, *their drinking might increase*, *so what was usually a controlled habit might become more using it as a coping strategy for the stresses…”* (GP3)
	*Social and cultural aspects*	*“You can go back to feeling like you were before you had babies and erm you know you’re out and about”* (M3)
		*“I think there’s just so much about that it’s acceptable for women to drink erm because they’ve got kids*. *‘Oh I’ve got to have a glass of wine you know the kids are driving me mad you know’*, *there’s that association I think is quite strong erm and I’ve always thought*, *found that on social media*. *There’s like a book ‘Hurrah for Gin’ erm you know there’s a lot of things about there’s probably more that promotes it for mothers than puts people off”* (M3)
		*“It was actually harder not to drink after having the baby because I’m suddenly feeling you know I just want to claw back a little bit of having a social life having a bit of a life*.*”* (MW1)
***Motives against drinking***		
	*Parental responsibilities*	*“My fear is what if I needed to go to the hospital in the middle of the night and I couldn’t drive to take him*.*”* (MW2)
		*“I think the big things modelling behaviour isn’t it*, *if your way of parenting is to sit in the pub and the kids just run around and you’re just socialising and getting drunk which let’s face it there’s loads of people that do that of a weekend don’t they*.*”* (MW1)
		*“…hangover was bad enough when you could stay in bed and get hydrated for the whole day*, *now having hangover when you have kids jumping on your head and requiring and still needing care and food and err it doesn’t sound like fun in the first place”* (P3)
		*“… the stigma of it like if you were to go out and see … a family having a meal … if you saw both of them drinking then I think it’s more frowned upon if it’s a woman because they’re not supposed to do that and I think a lot of its around like societies perception of you and your role so I think that would be it you don’t want to be seen as a bad mum”* (M6)
		*“In terms of erm opportunity to drink there’s probably going to be much more restricted*. *So I think I’ll probably will end up drinking much less than what I drank before like getting pregnant”* (P4)
		*“…I think at the end of the night when the baby’s gone to bed you feel like you’ve got a bit of your time… when you’ve got a baby*, *you can’t really go out and have a drink out … prior to having Olivia we might go up the village for a drink you can’t do any of that it’s just impossible so it’s like a way of still having a drink and having a relax but adapting it to what you’re actually able to do if that makes sense”* (M6)
	*Personal preference*	*“Personal choice*. *I think now there’s a big movement about veganism*, *being very*, *you know*, *body conscious*, *and being aware of what you eat and drink and organic*, *and so we have a lot of patients that are being very very healthy*, *almost extreme life styles*.*”* (GP1)
		*“I mean I’ve cut down on my drinking overall because my dad was erm alcoholic erm and he passed away”* (P5)

P = pregnant woman; M = mother; GP = general practitioner; MW = midwife

## Discussion

This study investigated the attitudes and experiences of pregnant and parenting women and healthcare professionals using multiple qualitative methods. The UK Chief Medical Officer recommends abstinence during pregnancy and many women comply, recognising the potential risks drinking poses to the fetus, the mother, and those around them. Nevertheless, low level drinking was viewed as acceptable by some women due to insufficient evidence concerning the damaging effects of alcohol use at low levels. Women and healthcare professionals agreed that drinking in moderation is acceptable once the baby has been born, but that excessive drinking can carry significant risks.

Although data from the different populations were analysed separately, they are reported together due to a high degree of similarity in the responses. Any differences in the views of pregnant women, mothers, and healthcare professionals have been highlighted. This close resemblance may be the result of many of the healthcare professionals having children themselves and thus providing a combination of perspectives as professionals and as mothers.

Women and healthcare professionals associated drinking while pregnant with high risk. Direct harm to the baby was identified including numerous developmental issues and birth defects. Excessive drinking was related to mental and physical health consequences to the mother, with psychological problems potentially arising even at lower level drinking (e.g., feeling shame and guilt). Any level of drinking was found to be a potential source of disruption in the family due to differing opinions between family members (e.g., intergenerational differences in opinion) about alcohol consumption and the responsibility of the mother carrying a child. This awareness of alcohol-related harm resembles previous research findings [41].

In line with previous research on motives for alcohol use [42], narratives demonstrated that pregnant women drink as a coping strategy or due to an existing alcohol use problem. Alcohol was also considered to have a strong social and cultural aspect, being part of everyday life and celebration [43]. Becoming pregnant is a significant life transition involving great changes, which women may find difficult to adapt to. By contrast, the damaging effects of alcohol to the fetus, the prospect of feeling guilty, and social stigma may stop some women from drinking.

Women’s uncertainty regarding whether low-level drinking during pregnancy is acceptable has also been suggested by previous research [41, 44]. Although women were aware of the potential damaging effects of alcohol use during pregnancy, confusing advice from formal (health care professionals featured in the media, government guidelines and policies) and informal (friends, family, and the internet) resources, and the absence of clear evidence regarding safe levels of drinking polarised women’s attitudes [41]. Some chose ‘to be on the safe side’ to avoid any potential harm to the baby by remaining abstinent. Others advocated the safety of some minimal drinking and considered abstinence guidelines stigmatising and patronising, which may lead to underreporting of consumption [45]. This was reflected in the inconsistent reporting found when comparing some of the quantitative and qualitative responses. Current guidelines promote complete abstinence justified by the paucity of evidence for a safe amount [46]. For women to follow these guidelines and change their alcohol-related attitude and behaviour, advice needs to be clear and consistent providing specific details of the potential harms low level drinking may cause [9, 44]. Importantly, midwives should be trained to provide alcohol advice appropriate to drinking level and given sufficient time to do this [46]. These needs are echoed by recent evidence which found that UK midwives may not know the source of current recommendations for abstention during pregnancy [47].

During motherhood, low-level drinking was reported as acceptable but drinking to excess was not, due to its potentially serious consequences while carrying out parental responsibilities. However, the definition of the differing levels was unclear, leading to contradictions in findings when comparing the qualitative narratives and quantitative responses. These conflicts suggest that women underreport their level of alcohol use, perhaps as an implicit or explicit way to protect their own or others’ perceptions of them. Under reporting is observed by previous research, due to fear of stigmatisation and disclosure consequences [24], and social desirability [25]. Moreover, all participants viewed drinking levels in extremes considering only having the occasional drink on one end and excessive/problematic drinking on the other. This suggests a lack of awareness of ‘grey area drinking’, a moderate level of consumption (drinking seven or fewer drinks weekly and three or fewer daily, but consuming more than one drink at least on one day) with potential harms, such as increasing level of alcohol use, development of alcohol dependence, job loss, and interpersonal relationships [48]. However, some mothers did recognise that development of problematic drinking could occur.

Although UK Chief Medical Officer guidelines recommend limiting weekly alcohol consumption to 14 units [17], it also warns about the increased risk of any regular alcohol use with regards to heart disease and breast cancer [49], some of which may be typically female diseases. Additionally, guidelines during breastfeeding are significantly lower (“no more than one or two units of alcohol once or twice a week”; [40]) due to the additional dose-dependent risks of alcohol use to the offspring. Overall, participants recognised some of the risks excessive drinking poses [50] and these were consistent with existing literature (e.g., to mothers’ and children’s mental and physical health [16, 18, 19], to mothers’ parenting ability [51, 52], and to relationships [53, 54]). However, there was no mention of any specific physical health consequence of regular alcohol consumption which was not associated with clinically problematic drinking [17]. To enable positive childhoods, it is important that we facilitate good health and well-being in parents. This includes supporting healthy lifestyle choices, such as low-level alcohol use. Additionally, there should be specific focus on how to better communicate potential risks around drinking while breastfeeding [55], to help mothers adhere to guidelines.

Reflecting existing evidence [12, 13, 56], narratives regarding why women with children do and do not consume alcohol demonstrate that the transition to motherhood may indeed be a protective factor against alcohol use/abuse (at least during early postpartum periods). According to women’s reports, alcohol use stopped or decreased in order to fulfil parental responsibilities, such as breastfeeding, co-sleeping, looking after the child/children, and modelling appropriate health behaviours. Nevertheless, alcohol may also be used to cope with a life transition that can potentially be extremely stressful [12]. The participants of this study described motherhood as stressful and challenging with potential negative emotions and mental struggles which may exacerbate alcohol use rather than reduce it. Although women carrying their first child expected to start drinking less or not at all once they become mothers, due to the lack of opportunity to go out and socialise, mothers reported a change in drinking habits by drinking at home at the end of a stressful day. This type of drinking to cope in the home poses its own risks, such as developing conditioned tolerance to the effects of alcohol consumed in a familiar environment where one feels less intoxicated, increasing the tendency to drink more [57]. Those who chose to avoid alcohol focused on the social norms dictating that mothers shouldn’t drink due to parental responsibilities [12]. In contrast, those who used alcohol to cope are strongly influenced by the narrative, widely endorsed in social and the general media, that drinking to cope with the stresses of motherhood is acceptable, common, and funny. More research is necessary to understand the impact of such messages to vulnerable women.

Despite efforts to recruit participants with different economic and social positions, through visiting children (community) centres in a range of neighbourhoods, we were unable to recruit women from lower socioeconomic backgrounds. Convenience sampling resulted in the recruitment of primarily white, middle-class women aged 30+, the population most associated with alcohol use in pregnancy [7]. However, it is important to note that alcohol harm is more likely to be experienced in those in more disadvantaged circumstances [58] and further research is necessary to capture their views and attitudes.

Although contradictions in relation to alcohol use found in the narratives reflect a certain degree of potential underreporting, participants of this study primarily reported good levels of mental health and low levels of alcohol use. Understanding such types of alcohol-related behaviours is important because it contributes to preventative work. But data from a more diverse sample would be beneficial, providing information on a wider range of behaviours related to alcohol use and their social and environmental influences. This will increase the information power [36] of future work in this area.

Acquiring the view of a range of healthcare professionals provided a more comprehensive picture of alcohol consumption in motherhood. However, due to work commitments, we provided the opportunity for healthcare professionals to take part in the research through a focus group or individual interviews. While focus groups and individual interviews are independent data collection methods and may be challenging to combine the resulting data, this combination can be advantageous in generating complementary views of a given phenomenon [59]. The data sets obtained in this study were not assumed to be equivalent but were judged to be adequate for integration in providing a better understanding of the views of healthcare professionals. While this type of triangulation of the data may be useful and practical, identification of its value and trustworthiness needs further investigation [59]. Additional research is necessary to provide a more comprehensive exploration of healthcare professionals’ perspectives on alcohol-related advice to pregnant and parenting mothers.

### Conclusion

Drinking during pregnancy and motherhood is associated with unique and significant primary and secondary harms to the mother and fetus/child, respectively, as well as the wider family. This study highlights some degree of inconsistency in women’s attitudes on drinking during pregnancy and motherhood. Additionally, findings support previous work that mothers may underreport their drinking behaviour, that women may not accurately recognise how much they drink, or how harmful alcohol use can be at non-dependent drinking levels. Moving forward, clear and consistent advice, given in a compassionate way, is needed around drinking during pregnancy, breastfeeding, and motherhood in order to motivate and support change in maternal drinking behaviours.

## Supporting information

S1 Fig Alcohol consumption-related views and experience of pregnant women, mothers, and healthcare professionals during pregnancy. Themes in black illustrate views of pregnant women and mothers overall with those of the healthcare professionals, and them in white illustrate where they differ.
(TIF)

S2 Fig Alcohol consumption-related views and experiences of pregnant women, mothers, and healthcare professionals during motherhood. (TIF)

S1 Table Demographic information. P = pregnant woman; M = mother; GP = general practitioner; MW = midwife; SMP = substance misuse practitioner.
(DOCX)

S2 Table Birth experience, alcohol use, and mental health. P = pregnant woman; M = mother. AUDIT score of 1–6 indicates low-risk drinking; AUDIT score of 7 or more in women indicates a strong likelihood of hazardous or harmful drinking; AUDIT score of 20 or above suggests alcohol dependence.
(DOCX)

S1 File Interview schedule with pregnant women and mums. (DOCX)

S2 File Interview schedule with health care professionals. (DOCX)

S3 File COREQ checklist. (DOCX)
