# Supplementary figures and images for: Alcohol use during pregnancy and motherhood: Attitudes and experiences of pregnant women, mothers, and healthcare professionals
Source: PLoS One. 2022 Dec 1;17(12):e0275609. doi: 10.1371/journal.pone.0275609 (PMC9714863; doi:10.1371/journal.pone.0275609)

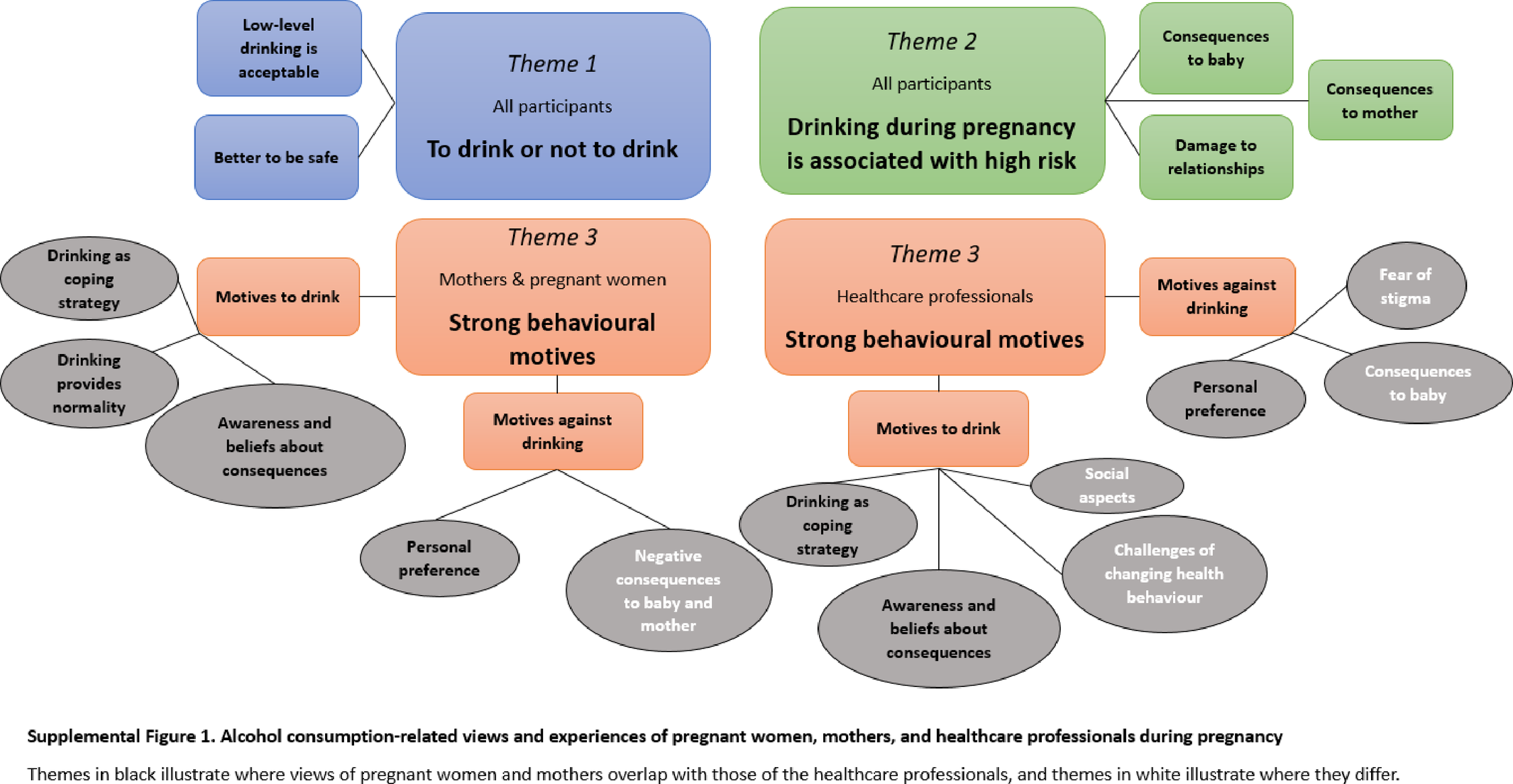

Supplement: S1 Fig — Themes in black illustrate views of pregnant women and mothers overall with those of the healthcare professionals, and them in white illustrate where they differ. (TIF) [file pone.0275609.s001.tif]

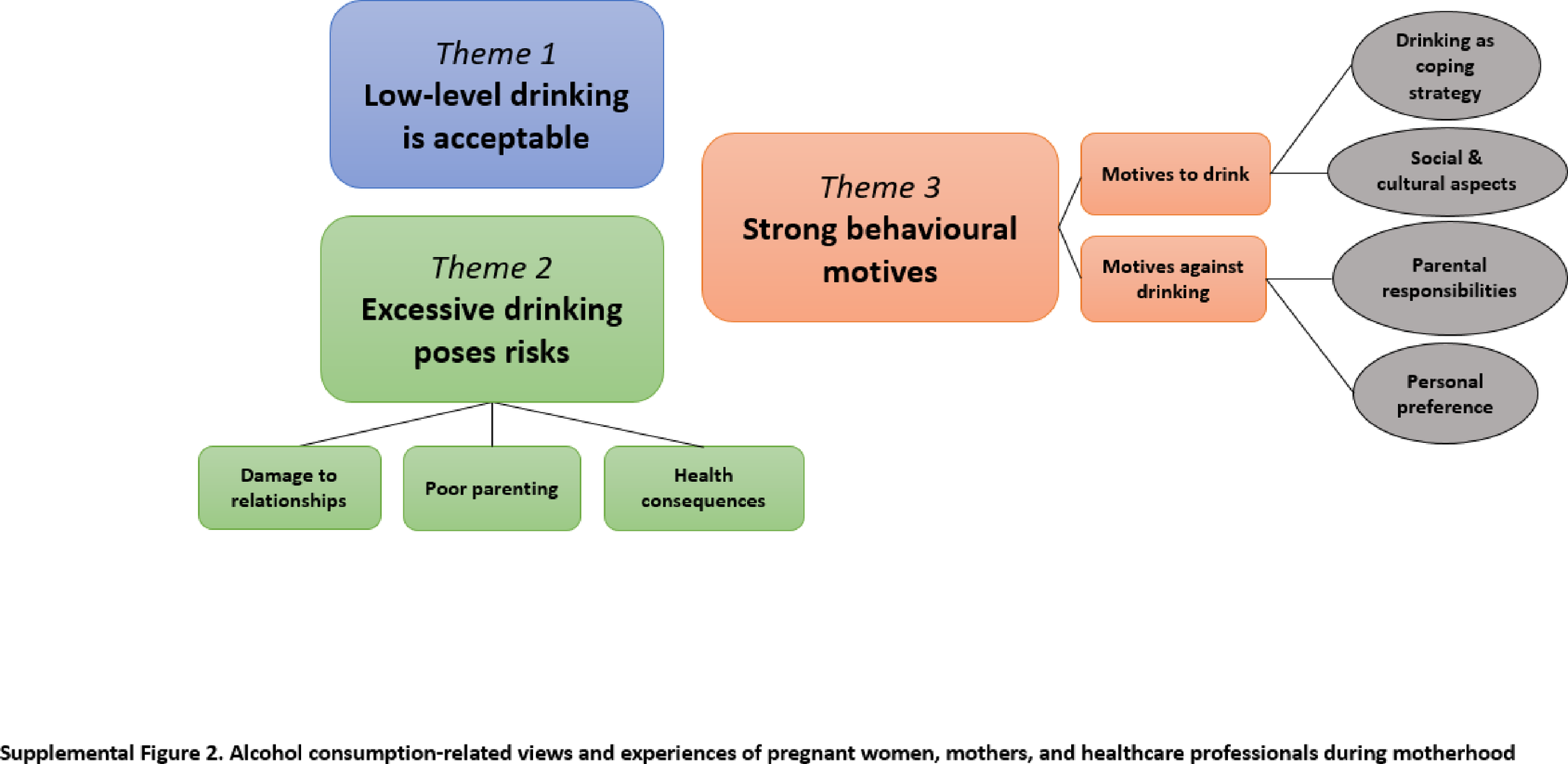

Supplement: S2 Fig — (TIF) [file pone.0275609.s002.tif]

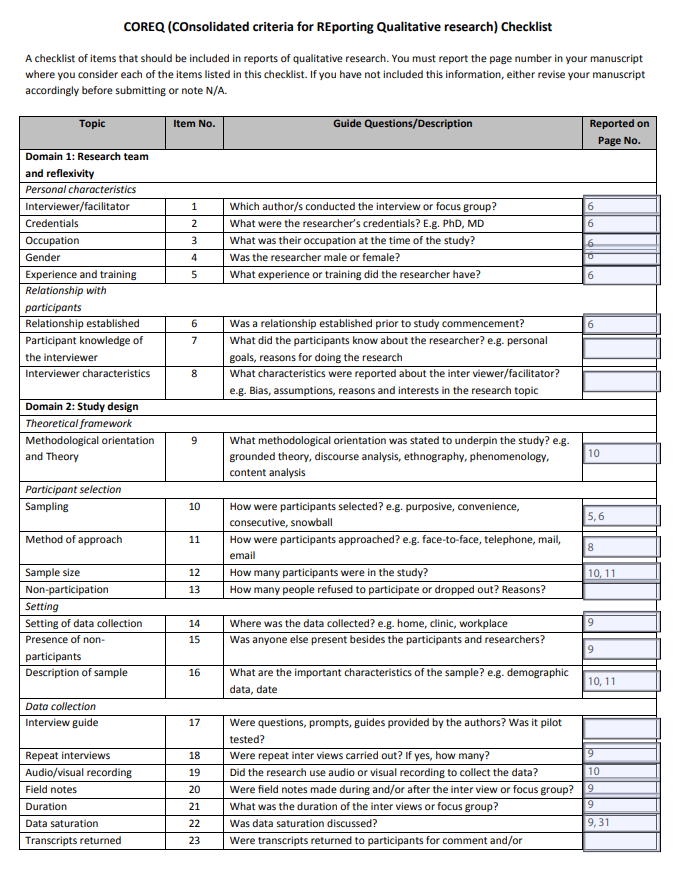


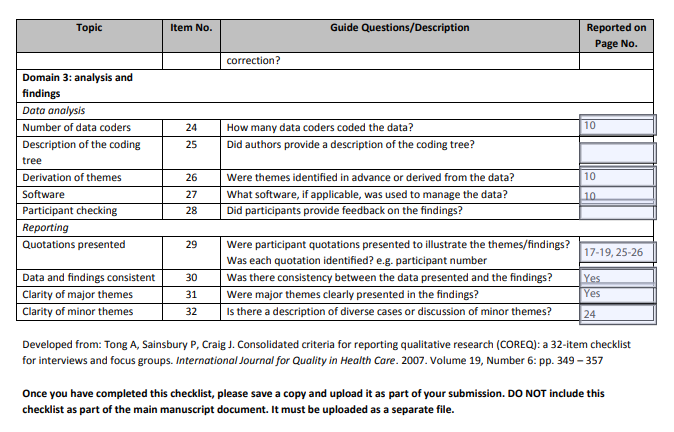

Supplement: S3 File — (DOCX) [file pone.0275609.s007.docx]
