# Supplementary material for: Alcohol use during pregnancy and motherhood: Attitudes and experiences of pregnant women, mothers, and healthcare professionals
Source: PLoS One. 2022 Dec 1;17(12):e0275609. doi: 10.1371/journal.pone.0275609 (PMC9714863; doi:10.1371/journal.pone.0275609)
Supplement: S1 Table — P = pregnant woman; M = mother; GP = general practitioner; MW = midwife; SMP = substance misuse practitioner. (DOCX) [file pone.0275609.s003.docx]

**Supplemental Table 1. Demographic information**

| **Participant** | **Age** | **Ethnicity** | **Marital status** | **Highest education level** | **Occupation** | **Annual household income** | **number of children** | **Age of children** |
| --- | --- | --- | --- | --- | --- | --- | --- | --- |
| **Pregnant women (N=6)** | | | | | | | | |
| P1 | 18-25 | White British | Single | Secondary school | N/A | <£20,000 | 2 | 4&6 years |
| P2 | 30-35 | Black British | Married/co-habiting | Bachelor’s degree | N/A | £56,000-£70,000 | 1 | 3 years |
| P3 | 30-35 | White European | Married/co-habiting | Doctorate degree | N/A | £31,000-£40,000 | 1 | 3 years |
| P4 | 30-35 | White British | Married/co-habiting | Doctorate degree | N/A | £56,000-£70,000 | 0 | - |
| P5 | 36-39 | White British | Married/co-habiting | Bachelor’s degree | N/A | £<£20,000 | 0 | - |
| P6 | 30-35 | White European | Married/co-habiting | Master’s degree | N/A | £41,000-£55,000 | 1 | 3 years |
| **Mothers (N=8)** | | | | | | | | |
| M1 | 30-35 | White British | Married/co-habiting | Master’s degree | N/A | £86,000-£99,000 | 1 | Under 1 |
| M2 | 36-39 | White British | Married/co-habiting | Bachelor’s degree | N/A | £71,000-£85,000 | 2 | 3 years,  Under 1 |
| M3 | 40-45 | White British | Married/co-habiting | Bachelor’s degree | N/A | £41,000-£55,000 | 3 | 5&7 years,  Under 1 |
| M4 | 30-35 | White British | Married/co-habiting | Secondary school | N/A | £21,000-£30,000 | 2 | 1&3 years |
| M5 | 40-45 | White British | Married/co-habiting | Doctorate degree | N/A | >£100,000 | 3 | 8, 10, & 13 years |
| M6 | 30-35 | White British | Married/co-habiting | Master’s degree | N/A | £71,000-£85,000 | 1 | 2 years |
| M7 | 26-29 | White British | Married/co-habiting | Bachelor’s degree | N/A | £31,000-£40,000 | 1 | Under 1 |
| M8 | 30-35 | Black other | Married/co-habiting | Bachelor’s degree | N/A | <£20,000 | 2 | 5&7 years |
| **Healthcare professionals (N=7)** | | | | | | | | |
| GP1 | 40-45 | Asian British | N/A | N/A | General practitioner (for 13 years) | N/A | 2 | 3&10 years |
| GP2 | 46+ | White British (male) | N/A | N/A | General practitioner (for 29 years) | N/A | 2 | 18&22 years |
| GP3 | 46+ | White South African | N/A | N/A | General practitioner | N/A | 0 | - |
| MW1 | 30-35 | White British | N/A | N/A | Midwife (for 6 years) | N/A | 2 | 3 years,  under 1 |
| MW2 | 36-39 | White British | N/A | N/A | Midwife (for 11 years) | N/A | 1 | - |
| MW3 | 26-29 | White British | N/A | N/A | Midwife (for 8 years) | N/A | 1 | 2 years |
| SMP1 | 18-25 | White British | N/A | N/A | Substance misuse practitioner (for 18 months) | N/A | 0 | - |

P=pregnant woman; M=mother; GP=general practitioner; MW=midwife; SMP=substance misuse practitioner.
