# Supplementary material for: Alcohol use during pregnancy and motherhood: Attitudes and experiences of pregnant women, mothers, and healthcare professionals
Source: PLoS One. 2022 Dec 1;17(12):e0275609. doi: 10.1371/journal.pone.0275609 (PMC9714863; doi:10.1371/journal.pone.0275609)
Supplement: S2 Table — P = pregnant woman; M = mother. AUDIT score of 1–6 indicates low-risk drinking; AUDIT score of 7 or more in women indicates a strong likelihood of hazardous or harmful drinking; AUDIT score of 20 or above suggests alcohol dependence. (DOCX) [file pone.0275609.s004.docx]

**Supplemental Table 2. Birth experience, alcohol use, and mental health**

| **Participant** | **Traumatic birth experience** | **Traumatic life experience** | **Any alcohol consumption** | **Type of drink consumed** | **Amount of drink consumed** | **Frequency of alcohol consumption** | **AUDIT** | **Depression (PHQ-2)** | **Anxiety (GAD 2)** |
| --- | --- | --- | --- | --- | --- | --- | --- | --- | --- |
| **Pregnant women (N=6)** | | | | | | | | | |
| P1 | No | No | No (at the time of interview) | - | - | - | Hazardous drinker  (first 5 months of pregnancy) | Positive screen | Negative screen |
| P2 | No | No | Yes | Wine | 1x 175 ml | Occasionally | Unclear responses | Negative screen | Negative screen |
| P3 | No | No | No | - | - | - | Non-drinker | Negative screen | Negative screen |
| P4 | - | Yes | No | - | - | - | Non-drinker | Negative screen | Negative screen |
| P5 | - | Yes | No | - | - | - | Non-drinker | Negative screen | Negative screen |
| P6 | No | Yes | No | - | - | - | Non-drinker | Negative screen | Negative screen |
| **Mothers (N=8)** | | | | | | | | | |
| M1 motherhood | Yes | No | Yes | Wine  Lager | 3x 175 ml  1x pint of lager | weekly | Low-risk drinker | Negative screen | Negative screen |
| *pregnancy* |  |  | *Yes* | *Lager* | *1 or 2* | *once or twice during pregnancy* |  |  |  |
| M2 motherhood | No | No | Yes | Wine | 1x 175ml | weekly | Low-risk drinker | Negative screen | Negative screen |
| *pregnancy* |  |  | *Yes* | *Wine*  *Spirits* | *1 or 2* | *monthly* |  |  |  |
| M3 motherhood | Yes | No | Yes | Lager | 4x pint of lager | weekly | Low-risk drinker | Negative screen | Negative screen |
| pregnancy |  |  | No | - | - | - |  |  |  |
| M4 motherhood | No | No | Yes | Wine | 10x 175ml | weekly | Hazardous drinker | Negative screen | Negative screen |
| *pregnancy* |  |  | *Yes* | *Wine* | *1 or 2* | *monthly* |  |  |  |
| M5 motherhood | Yes | No | Yes | Wine | 4x 250ml | weekly | Low-risk drinker | Negative screen | Negative screen |
| *pregnancy* |  |  | *No* | *-* | *-* | *-* |  |  |  |
| M6 motherhood | No | No | Yes | Wine  Spirits | 2x 175 ml  7x 35ml | weekly  weekly | Low-risk drinker | Negative screen | Negative screen |
| *pregnancy* |  |  | *Yes* | *Wine* | *1 or 2* | *once or twice during pregnancy* |  |  |  |
| M7 motherhood | Yes | No | Yes | Wine | 3-4x 175ml | weekly | Low-risk drinker | Negative screen | Negative screen |
| *pregnancy* |  |  | *No* | *-* | *-* | *-* |  |  |  |
| M8 motherhood | No | No | No | - | - | - | Non-drinker | Negative screen | Negative screen |
| *pregnancy* |  |  | *No* | *-* | *-* | *-* |  |  |  |

P=pregnant woman; M=mother. AUDIT score of 1-6 indicates low-risk drinking; AUDIT score of 7 or more in women indicates a strong likelihood of hazardous or harmful drinking; AUDIT score of 20 or above suggests alcohol dependence.
