# Supplementary material for: Alcohol use during pregnancy and motherhood: Attitudes and experiences of pregnant women, mothers, and healthcare professionals
Source: PLoS One. 2022 Dec 1;17(12):e0275609. doi: 10.1371/journal.pone.0275609 (PMC9714863; doi:10.1371/journal.pone.0275609)
Supplement: S2 File — (DOCX) [file pone.0275609.s006.docx]

**Interview questions with healthcare professionals**

1. **Drinking during pregnancy**
   1. What are your views on drinking alcohol during pregnancy?
   2. What do you think the benefits and/or risks are of drinking alcohol during pregnancy?
   3. Where do you think the majority of women get their information from about drinking alcohol during pregnancy?
   4. What do you think are the reasons for women drinking alcohol during pregnancy?
   5. What do you think are the reasons for women not drinking alcohol during pregnancy?
   6. What do you think might be the short or long-term effects of drinking alcohol during pregnancy? This could be effects to you, your child or other family members/friends. (as this is a complex question, ensure that it is fully answered by breaking it up if necessary)
2. **Drinking during early motherhood (when child(ren) are below school age)**
   1. What are your views on drinking alcohol when your children are young?
   2. What do you think the benefits and/or risks are of drinking alcohol during early motherhood?
   3. Where do you think the majority of women get their information from about drinking alcohol during early motherhood?
   4. What do you think are the reasons for women drinking alcohol during early motherhood?
   5. What do you think are the reasons for women not drinking alcohol during early motherhood?
   6. What do you think might be the short or long-term effects of drinking alcohol during early motherhood? This could be effects to you, your child or other family members/friends. (as this is a complex question, ensure that it is fully answered by breaking it up if necessary)
